# Valorizing Carbide Slag as Cement-Compatible CaCO_3_ Suspension via Pretreatment-Controlled Carbonation

**DOI:** 10.3390/ma19173765

**Published:** 2026-09-04

**Authors:** Jiahui Zhu, Zanqun Liu, Yu Cui, Haitao Chen, Liping Guo, Geert De Schutter

**Affiliations:** 1 School of Civil Engineering, Central South University, Changsha 410075, China; jiahui.zhu@ugent.be (J.Z.);; 2 Magnel-Vandepitte Laboratory, Department of Structural Engineering and Building Materials, Ghent University, Zwijnaarde, 9052 Ghent, Belgium; 3 School of Materials Science & Engineering, Southeast University, Nanjing 211189, China

**Keywords:** CaCO_3_ suspension, carbide slag, Carbonation control, interfacial compatibility, nanoindentation

## Abstract

Calcium carbonate (CaCO_3_) suspensions derived from industrial calcium sources offer a viable route for low-carbon cement systems, yet their effectiveness is frequently constrained by limited interfacial compatibility with hydration products. In this study, carbide slag was carbonated to prepare CaCO_3_ suspensions, and an integrated pretreatment–carbonation route combining acid washing, wet milling, and triethanolamine-assisted crystallization regulation was employed to modify the carbonation products. Following the integrated treatment, the MgO and SO_3_ contents decreased from 0.93% and 1.52% in CS-CaCS to 0.33% and 0.51% in MCS-CaCS, respectively, accompanied by reduced coarse agglomeration and improved apparent wetting behavior. At a carbonate dosage of 15%, the 28-day compressive strength increased from 55.7 MPa for CS-CaCS to 76.8 MPa for MCS-CaCS, corresponding to an improvement of 37.9%. Nanoindentation further showed that the mean hardness and reduced modulus increased from 1.81 to 6.18 GPa and from 50.0 to 74.7 GPa, respectively. The performance recovery is attributed to the concurrent effects of suppressed impurity inheritance, reduced agglomeration, enhanced carbonate participation in hydration, and improved local particle–hydrate organization. These findings demonstrate the potential of the integrated route for converting carbide slag into a more cement-compatible CaCO_3_ suspension.

## 1. Introduction

The transition of cement-based materials toward low-carbon targets and higher functional integration has intensified research interest in microstructural optimization and composite design [1,2,3,4,5,6]. Against this backdrop, nano-sized calcium carbonate (nano-CaCO_3_) has been widely studied for its ability to regulate hydration-phase evolution [7,8]. It promotes heterogeneous nucleation of calcium silicate hydrate (C–S–H) and facilitates the formation of carbonate-bearing AFm (alumina, ferric oxide, monosulfate) phases, thereby modifying hydration pathways and contributing to matrix densification [9,10,11,12]. In addition, its nanoscale dimensions enable effective void filling, contributing to strength enhancement and microstructural stability [13,14,15].

Despite its potential, the engineering use of nano-CaCO_3_ is hindered by dispersion instability and limited interfacial efficiency. Dry-processed nanoparticles readily agglomerate in highly alkaline pore solutions, markedly reducing their effective surface area and limiting their interaction with hydration phases [16,17]. Although dispersion strategies such as nano-coating can mitigate particle agglomeration, the hydration response remains sensitive to the resulting particle distribution and agglomeration state [18,19]. These limitations become more pronounced at high dosages and may eventually offset or even reverse the expected performance gains.

To circumvent the limitations of powder-based nano-CaCO_3_, our previous work introduced a suspension-based approach in which CaCO_3_ nanoparticles were delivered as an in-situ carbonated calcium suspension (CaCS) synthesized by aqueous carbonation of Ca(OH)_2_. This aqueous-phase route ensures high dispersion and wettability without surface modification, enabling continuous interfacial contact with hydration products such as C–S–H [20,21].

However, this strategy is fundamentally constrained by the carbon intensity associated with Ca(OH)_2_ production. Industrial Ca(OH)_2_ is obtained through the calcination–hydration route (CaCO_3_ → CaO → Ca(OH)_2_), where limestone decarbonation alone releases approximately 0.7–0.8 t CO_2_ per ton of CaO, and the overall process typically reaches 1.1–1.4 t CO_2_ per ton when fuel consumption is included [22,23,24]. Consequently, the use of virgin Ca(OH)_2_ as a carbonation precursor partly offsets the environmental benefit of subsequent CO_2_ uptake because substantial process- and fuel-related emissions arise during lime production [25,26].

In this context, carbide slag (CS)—an acetylene-industry by-product rich in Ca(OH)_2_—has emerged as a potentially low-carbon calcium precursor. Previous studies have mainly utilized CS as a calcium-bearing raw material for clinker production [27] or as an alkaline activator in solid-waste cementitious materials [28]. More recently, Yang et al. employed selective calcium extraction and additive-assisted precipitation to improve the purity and regulate the particle size and polymorphism of carbide-slag-derived CaCO_3_ [29]. Chindaprasirt et al. demonstrated that triethanolamine modified the morphology and phase composition of CaCO_3_ precipitated from an aqueous calcium carbide residue solution, although the resulting product was evaluated as a polymer filler rather than in cementitious systems [30]. Shen et al. further showed that the assembly conditions strongly affected the size, morphology, and aggregation state of CaCO_3_ particles produced from carbide slag [31]. More directly, Xu et al. reported that carbonation-treated carbide slag could improve the bulk performance of blended cement paste [32]. However, how pretreatment-dependent carbonate formation affects the hydration participation and local mechanical response of CS-derived CaCO_3_ remains insufficiently understood.

Against this background, the present study proposes an integrated pretreatment–carbonation strategy to regulate the formation pathway of CS-derived CaCO_3_. The resulting in-situ CaCO_3_ suspensions are examined using a multiscale framework integrating hydration phase evolution, microstructural characteristics, and local micromechanical response. This pathway-oriented approach provides insight into how carbonation regulation influences the structural role of waste-derived CaCO_3_ in cementitious systems and informs the development of calcium-rich industrial residues as cement-compatible additives.

## 2. Methodology

### 2.1. Raw Materials

**Figure 1:**
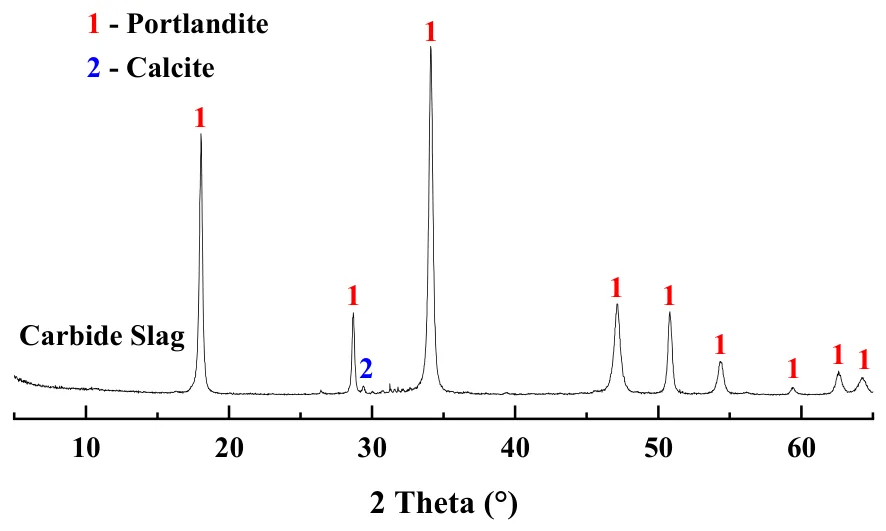
X-ray diffraction patterns of raw carbide slag.

**Figure 2:**
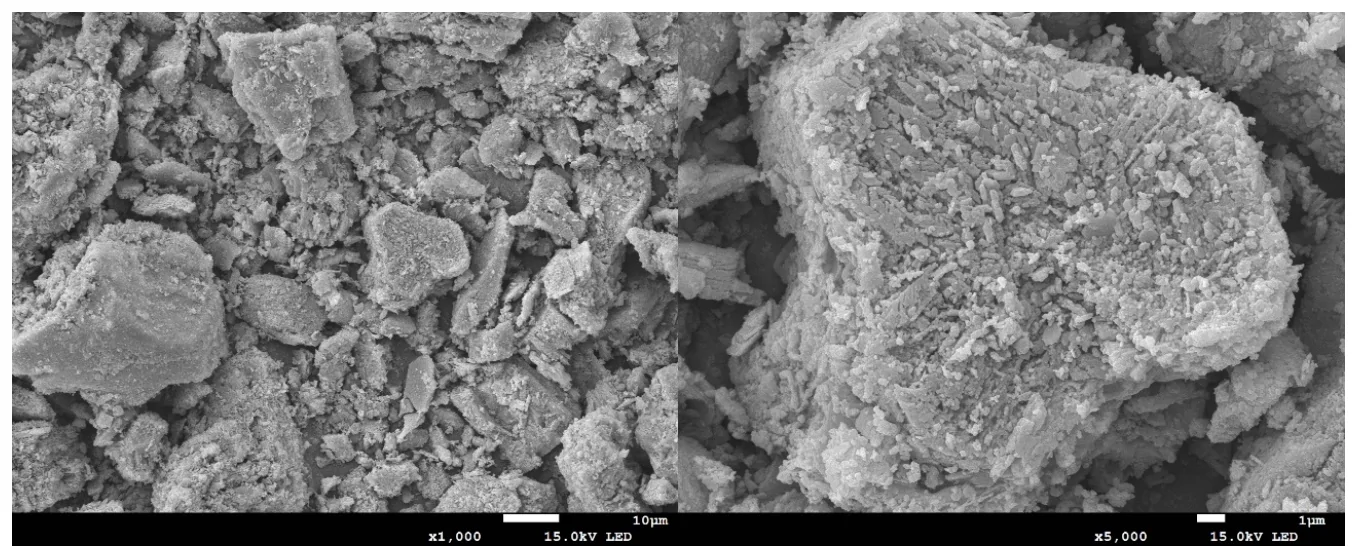
SEM image of raw carbide slag.

Industrial carbide slag (CS), obtained from acetylene production, was used as the calcium-rich precursor for in-situ carbonation. Analytical-grade Ca(OH)_2_ (CH) was employed as a chemically defined reference calcium source. The chemical and mineral compositions of CS and Portland cement are summarized in Table 1, and the XRD pattern and morphological features of raw CS are shown in Figure 1 and Figure 2. Citric acid monohydrate (C_6_H_8_O_7_·H_2_O, ≥99.5%) was used for acid leaching of CS, and triethanolamine (TEA, C_6_H_15_NO_3_, ≥99%) was used as a crystallization modifier during carbonation.

The CaO content determined by XRF represents the total Ca content expressed on an oxide-equivalent basis and should not be interpreted as the amount of free CaO or as the mineral-phase purity of CS. Because H and C are not represented in the reported XRF oxide composition, subtracting the CaO value from 100% does not provide the impurity fraction of CS. The phase composition was therefore determined separately by XRD–Rietveld refinement, which identified portlandite as the predominant crystalline phase in raw CS (97.3 wt%), together with a minor calcite fraction (2.6 wt%).

### 2.2. Sample Preparation

#### 2.2.1. Preparation of Carbonated Calcium Suspensions

A unified wet-carbonation procedure was used to prepare three carbonated calcium suspension systems: CH-CaCS from analytical-grade Ca(OH)_2_, CS-CaCS from untreated carbide slag, and MCS-CaCS from pretreated carbide slag. CH-CaCS and CS-CaCS were prepared by direct carbonation, whereas MCS-CaCS was produced using an integrated route comprising carbide-slag pretreatment followed by TEA-assisted carbonation.

(1) Pretreatment of carbide slag

For the preparation of MCS-CaCS, dried raw carbide slag was mixed with 0.1 mol/L citric acid solution at a solid-to-liquid ratio of 1:10 (g/mL) and stirred for 20 min at room temperature. The treated solid was separated, washed three times with deionized water, and dried at 60 °C. The acid-washed carbide slag was subsequently dispersed in deionized water to obtain suspensions with solid contents of approximately 10–33 wt%.

The suspension was transferred to a 500 mL zirconia jar of a QM-3SP2 planetary ball mill (Nanjing, China). Zirconia balls with diameters of 3 and 5 mm were used at a volume ratio of 1:1, and the ball-to-solid mass ratio was approximately 10:1. Wet milling was performed at 400 rpm. Suspensions with relatively low solid contents were milled in two 12 min cycles, whereas those with relatively high solid contents were milled in four 6 min cycles. A cooling interval of approximately 5 min was applied between successive cycles. After milling, the suspension was allowed to settle briefly and passed through a 2 mm sieve to separate the zirconia media. The resulting slurry was used directly for carbonation without intermediate drying to limit reagglomeration.

(2) Wet-carbonation procedure

**Figure 3:**
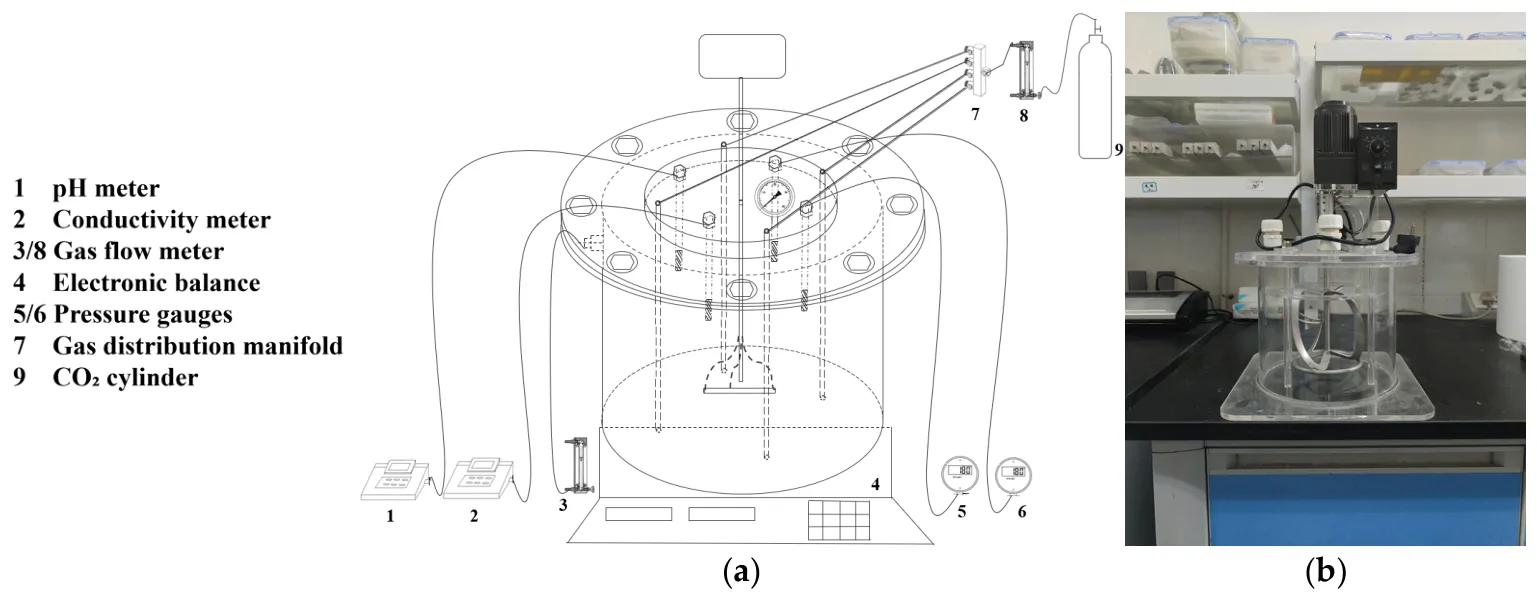
Wet carbonation equipment: (**a**) Design diagram. (**b**) Photograph of the carbonation vessel.

The wet-carbonation apparatus is shown in Figure 3. Predetermined amounts of the corresponding calcium-bearing precursor and deionized water (Table 2) were introduced into the carbonation vessel and predispersed by stirring for 5 min. CO_2_ was then supplied through a gas manifold connected to four submerged inlet tubes. The flow rate was maintained at approximately 60 mL/min per channel, corresponding to a total CO_2_ flow rate of approximately 240 mL/min. A helical impeller operated continuously to maintain suspension homogeneity and facilitate gas–liquid mass transfer. No crystallization modifier or surfactant was added to CH-CaCS or CS-CaCS. For MCS-CaCS, triethanolamine (TEA) was introduced at 2 g/L based on the total suspension volume to regulate CaCO_3_ nucleation and growth.

All carbonation experiments were conducted at atmospheric pressure and an initial laboratory temperature of approximately 20 °C. Reaction progress was monitored continuously using pH and conductivity probes immersed in the suspension. The operational endpoint was reached when the conductivity approached a stable plateau and the pH decreased to approximately 6.5. Depending on the precursor and solid content, carbonation generally required 20–40 min. Although no external heating was applied, the suspension temperature increased to approximately 40 °C during carbonation. After CO_2_ injection was terminated, the suspension was allowed to stand for 3 h to cool and stabilize before cement-paste preparation.

The three treatment operations used for MCS-CaCS addressed different aspects of the untreated precursor. Acid washing was used to suppress the inheritance of soluble and weakly bound impurities, wet milling was applied to disintegrate precursor agglomerates, and TEA was introduced during carbonation to regulate carbonate nucleation and growth. These operations were implemented sequentially as an integrated treatment route.

#### 2.2.2. Cement Paste Mixing and Casting

Cement pastes were prepared at a constant water-to-binder ratio (w/b) of 0.35. For the CaCS-containing groups, the carbonated calcium suspension was used as the mixing liquid, whereas the PC reference was prepared with deionized water. The carbonate dosage (CD) was defined based on the stoichiometric conversion of Ca(OH)_2_ to CaCO_3_ to ensure comparable carbonate levels across different systems. The CD was calculated based on the stoichiometry of the carbonation reaction:

(1) Ca(OH)_2_ (74 g/mol) + CO_2_ (44 g/mol) → CaCO_3_ (100 g/mol) + H_2_ O (18 g/mol)

According to the reaction stoichiometry, 74 g of Ca(OH)_2_ reacts with 44 g of CO_2_ to form 100 g of CaCO_3_ and 18 g of H_2_O. The total mass increase of the suspension during complete carbonation is therefore determined by the mass of absorbed CO_2_. The reaction also generates H_2_O, which remains part of the suspension matrix. Thus, the total mass of suspension formed from *a* g of Ca(OH)_2_ and *b* g of deionized water is approximated by 74*a* + 18*b*.

The total CaCO_3_ formed is:

(2) M_CaCO3_ = 100a

When *x* grams of the carbonated suspension are added to the cement paste, the corresponding CaCO_3_ introduced is

(3) $$x\cdot (\frac{100a}{118a+74b})$$

Let ***c*** be the mass of cement. The CaCO_3_ dosage (CD) is calculated as:

(4) $$\mathrm{CD}\;=\;\frac{x\cdot (\frac{100a}{118a\;+\;74b})}{x\cdot (\frac{100a}{118a\;+\;74b})+c}$$

To simplify,

(5) $$\mathrm{CD}\;=\frac{100a\cdot x}{100a\cdot x+c\cdot (118a+74b)}$$

Three carbonate dosage levels (5%, 15%, and 25%) were investigated, and the corresponding mix proportions are summarized in Table 2. Mixing, casting, curing, and hydration termination procedures are described.

The CaCO_3_-containing suspension (CaCS) was first poured into the mixing bowl. After 30 s of pre-stirring to redisperse the suspension, cement was added. The paste was then mixed in three sequential steps: 90 s at low speed, 120 s at high speed, and a final 30 s at low speed.

After mixing, the fresh paste was cast into molds. The molds were sealed with plastic film and stored at 20 °C for 24 h. Upon demolding, the specimens were cured in a controlled environment at (20 ± 1 °C) and relative humidity >95% until the designated testing age.

(a) Mechanical strength specimens:

Cubic specimens with dimensions of 20 mm × 20 mm × 20 mm were prepared and cured under standard conditions for compressive strength testing.

(b) Microstructural and micromechanical specimens:

Specimens prepared using the same mix proportions were subjected to hydration termination at the target curing age using isopropanol. Samples were immersed for 1 day, followed by solution replacement and an additional 6 days of soaking, then vacuum-dried at room temperature for 7 days [18].

(i) Powdered samples:

Hydration-stopped samples were ground and used for phase identification and hydration analysis by XRD and TG.

(ii) Intact block samples:

Hydration-stopped blocks were used for microstructural observation (SEM) and nanoindentation testing.

### 2.3. Characterization Methods

#### 2.3.1. Macroscopic Mechanical Tests

*Compressive strength:* Compressive strength was evaluated using laboratory-scale cement-paste specimens rather than standard mortar specimens for conformity assessment. Cubic specimens with dimensions of 20 mm × 20 mm × 20 mm were tested at 3, 7, and 28 days using a WDW-100E universal testing machine (Jinan Fangyuan, Jinan, China) at a constant loading rate of 0.2 kN/s. The smaller specimens were adopted because of the limited volume of each carbonated suspension batch and to ensure that specimens within each comparison were prepared using the same suspension batch. All groups were prepared, cured, and tested under identical conditions. Three replicate specimens were tested for each mixture and curing age, and the results are reported as the mean ± standard deviation. Because the non-standard specimen geometry is subject to size effects, the measured strengths were used only for intergroup comparison and not for standards-based conformity assessment.

#### 2.3.2. Microstructural and Physicochemical Characterization

*Dynamic contact-angle measurement:* The apparent dynamic wetting behavior of the carbonated calcium suspensions was characterized using a KRÜSS DSA100 drop-shape analyzer (KRÜSS GmbH, Hamburg, Germany) with the sessile-drop method. A 2 μL droplet of each suspension was deposited onto a clean, flat, non-absorbing substrate, and the droplet profile was recorded continuously during spreading. The first analyzable image immediately after deposition was defined as 0 s, and the profiles obtained at 0 and 5 s were used for comparison. Each measurement was repeated at least three times under identical conditions.

*X-ray diffraction*: Prior to X-ray diffraction (XRD) analysis, the dried samples were gently ground into fine powders and thoroughly homogenized to minimize the effects of particle-size variation and uneven phase distribution. The powders were evenly packed into the sample holder to obtain a flat measurement surface. XRD patterns were collected using a Rigaku D/max III diffractometer (Tokyo, Japan) in continuous scanning mode over a 2θ range of 5–65° with a step size of 0.0167°. The phase contents of the raw materials were determined by Rietveld refinement. For the cement-paste samples, hydration-product evolution was evaluated semi-quantitatively by peak-area integration, with rutile TiO_2_ used as an external intensity reference [33].

*Thermogravimetric analysis*: Thermogravimetric and derivative thermogravimetric analyses were performed using a NETZSCH STA 449C simultaneous thermal analyzer (NETZSCH, Selb, Germany). Approximately 50 mg of each powdered sample was heated from 25 to 1050 °C at 10 °C/min under a nitrogen purge flow of 20 mL/min.

*Scanning electron microscopy*: Microstructural observations were conducted using a Nova NanoSEM 230 field-emission scanning electron microscope (FEI, Hillsboro, OR, USA) under vacuum. To preserve the original morphology of the hydration products and particle-contact features as far as possible, unpolished natural fracture surfaces were preferentially selected. Before observation, hydration-terminated and dried specimens were mounted on sample stubs and sputter-coated with a thin platinum layer to improve electrical conductivity and reduce charging. Secondary-electron images were subsequently acquired under vacuum at different magnifications.

*Inductively coupled plasma–optical emission spectrometry (ICP–OES):* The liquid samples were diluted as required before analysis. The concentrations of Ca, Si, S, Ba, Mg, Na, K, and other relevant elements were determined using a SPECTROBLUE SOP inductively coupled plasma–optical emission spectrometer (SPECTRO Analytical Instruments, Kleve, Germany).

*Nanoindentation Testing:* Nanoindentation was performed using a NanoTest platform (Micro Materials Ltd., Wrexham, UK) equipped with a Berkovich diamond indenter (nominal tip radius ≈ 50 nm) under ambient laboratory conditions. All measurements were conducted in load-controlled mode. The loading protocol consisted of an initial load of 0.05 mN, followed by a loading segment at 0.10 mN·s^−1^ up to a peak load of 2.00 mN. A 5 s dwell was held at maximum load to stabilize the contact, after which unloading proceeded at 0.10 mN·s^−1^. Thermal drift was corrected using a 30 s drift segment acquired at the end of each indent.

Twenty indents were performed per specimen following the pre-programmed array pattern in the instrument log. The array consisted of five columns with four indents per column, with spatial offsets of 10 μm (Z direction) and 15 μm (Y direction) between adjacent impressions. For each indent, the load–displacement (P–h) response and associated mechanical parameters were evaluated using the Oliver–Pharr method, with a power-law fit applied to the upper 20–100% of the unloading curve. The elastic (W_e_) and plastic (W_p_) work components were calculated from the integrated areas under the unloading and loading segments, respectively, using the NanoTest analysis module.

The raw output files generated from the nanoindentation tests contain two categories of essential information: (i) point-wise load–displacement (P–h) data and (ii) indentation-specific mechanical parameters.

To compare the local indentation responses of the three systems, one representative P–h curve was selected from each dataset according to the following criteria: the loading trajectory had to exhibit no fracture events, discontinuities, or plateau regions.

Mechanical parameters, in contrast, were extracted from the full indentation dataset as described below:

➢ Fundamental mechanical parameters

(i) Hardness (H, GPa): resistance to permanent penetration.
(ii) Reduced modulus ($E_{r}$, GPa): reflects the elastic stiffness of the material.
(iii) Residual depth ($h_{r}$): the indentation depth retained after unloading, reflecting the plastic component of deformation.
(iv) Maximum depth ($h_{\max}$): peak penetration depth during loading.
(v) Plastic and elastic work ($W_{p}$, $W_{e}$): irreversible and recoverable components of the total indentation work.

➢ Normalized and composite parameters

(i) Residual-depth ratio ($\frac{h_{r}}{h_{\max}}$): indicator of elastic recovery.
(ii) Plastic-work fraction ($\frac{W_{p}}{W_{p}+W_{e}}$): proportion of work dissipated plastically.
(iii) Hardness-to-modulus ratio ($\frac{H}{E_{r}}$): descriptor of the elastic–plastic balance and resistance to yielding.
(iv) Statistical descriptors (mean, standard deviation, and coefficient of variation): used to characterize the central tendency and variability of the local mechanical response.

ChatGPT (GPT-4o; OpenAI, San Francisco, CA, USA) was used solely to generate illustrative elements of the graphical abstract based on the authors’ original concepts and instructions, and the final image was reviewed and approved by the authors.

## 3. Results

### 3.1. Interfacial Incompatibility of CS-Derived CaCO_3_

At comparable CaCO_3_ contents, the CH-CaCS system exhibits a distinct mechanical response relative to the CS-CaCS system. Representative compressive strength results show that the CS-CaCS system consistently displays lower strength than the CH-CaCS counterpart, indicating that in-situ CaCO_3_ formation alone is insufficient to ensure mechanical enhancement.

**Figure 4:**
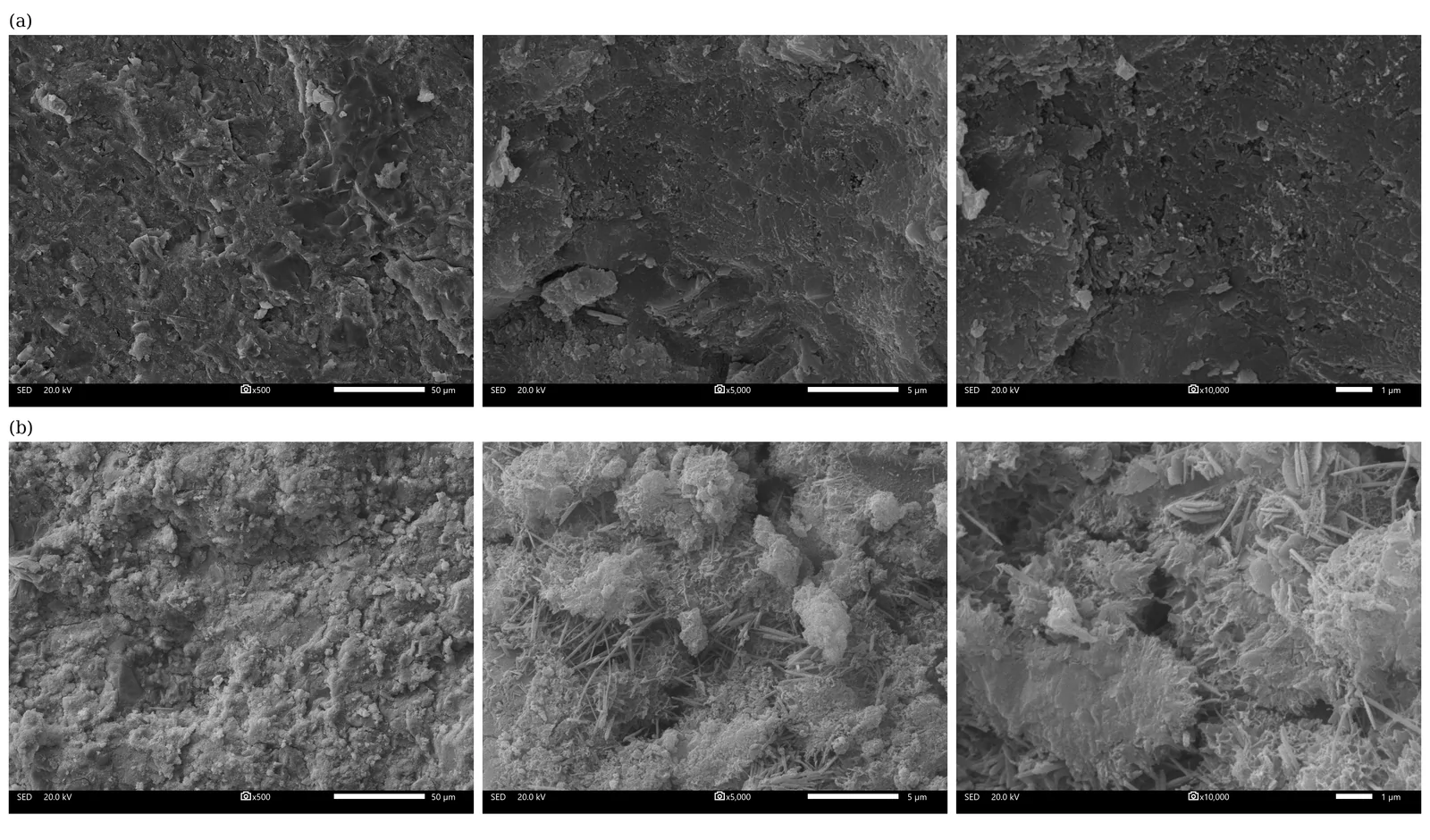
Microstructural morphology of cement pastes at 28 days of hydration: (**a**) CH-CaCS and (**b**) CS-CaCS.

**Figure 5:**
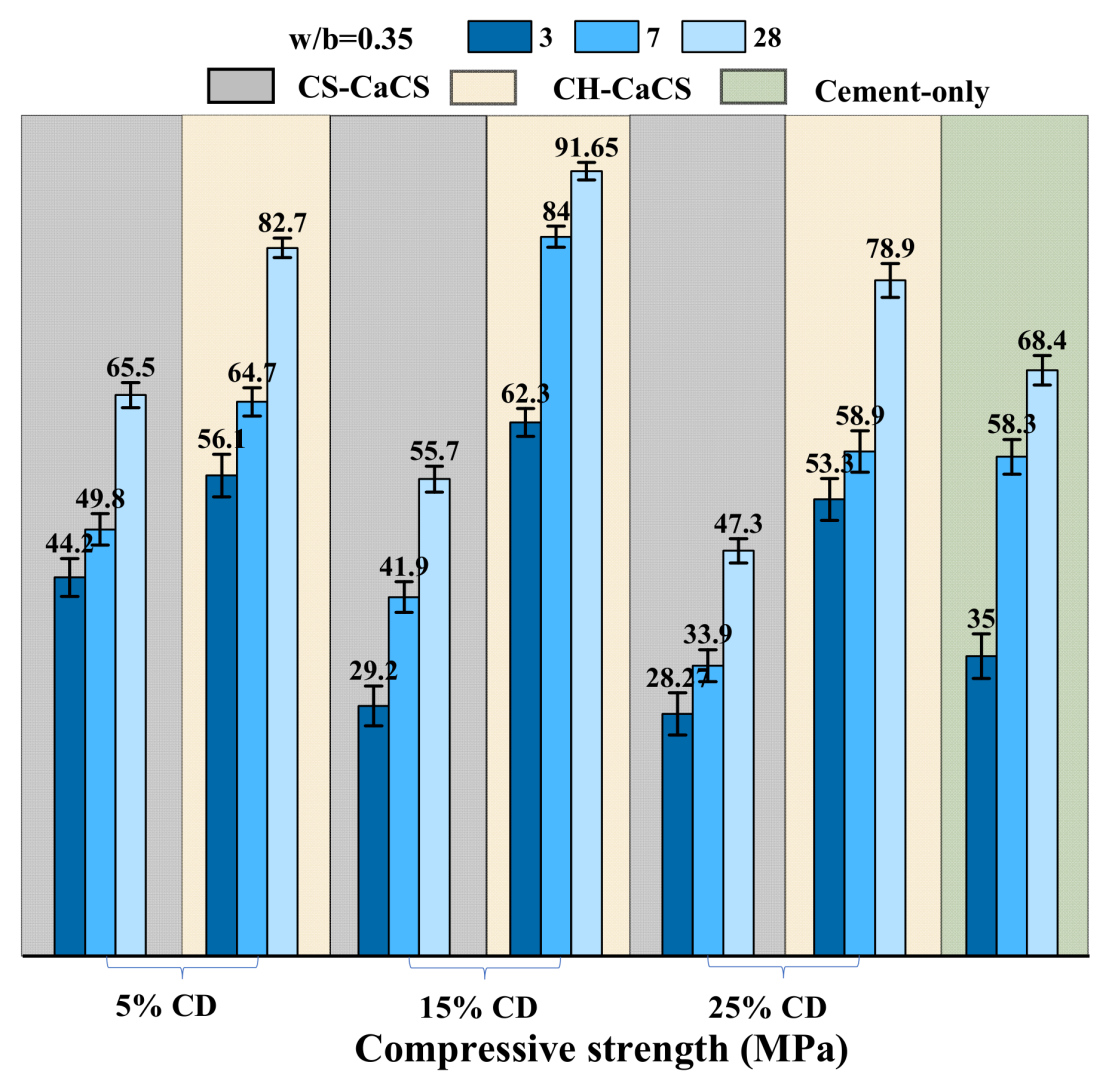
Compressive strength of cement pastes with CH-CaCS and CS-CaCS at various dosages and curing ages.

The higher-magnification SEM images reveal clear differences in the local microstructural morphology of the two systems (Figure 4). The CH-CaCS paste exhibits a relatively compact hydrate morphology, in which discrete carbonate-rich agglomerates are not readily distinguishable and the hydration products appear closely intergrown (Figure 4a). By contrast, the CS-CaCS paste contains coarse, irregular particle clusters surrounded by comparatively open regions, together with less continuous contact between the clustered particles and adjacent hydration products (Figure 4b). These recurring morphological features indicate less effective spatial incorporation of the CS-derived carbonation product into the hydrated matrix. These morphological differences are consistent with the lower compressive strengths of the CS-CaCS pastes compared with the CH-CaCS pastes at equivalent dosages and curing ages (Figure 5).

The mechanical implications of this interfacial contrast are already evident at the microscale. While detailed nanoindentation results are presented in Section 3.3.3, the CS-CaCS system exhibits a more heterogeneous and mechanically discontinuous local response, consistent with limited elastic load transfer across the CaCO_3_-cement interface.

### 3.2. Origin and Regulation of Interfacial Incompatibility in CS-Derived CaCO_3_

The inferior mechanical performance of the CS-CaCS system identified in Section 3.1 indicates a fundamental incompatibility between CS-derived CaCO_3_ and the cementitious environment. To rationally address this issue, it is necessary to trace the origin of the interfacial mismatch to the chemical and structural characteristics of CaCO_3_ formed from carbide slag, and to clarify how these characteristics deviate from those of CaCO_3_ generated from chemically defined calcium sources.

#### 3.2.1. Origin of Interfacial Incompatibility in CS-Derived CaCO_3_

(1) Impurity-mediated deviation of carbonate crystallography

**Figure 6:**
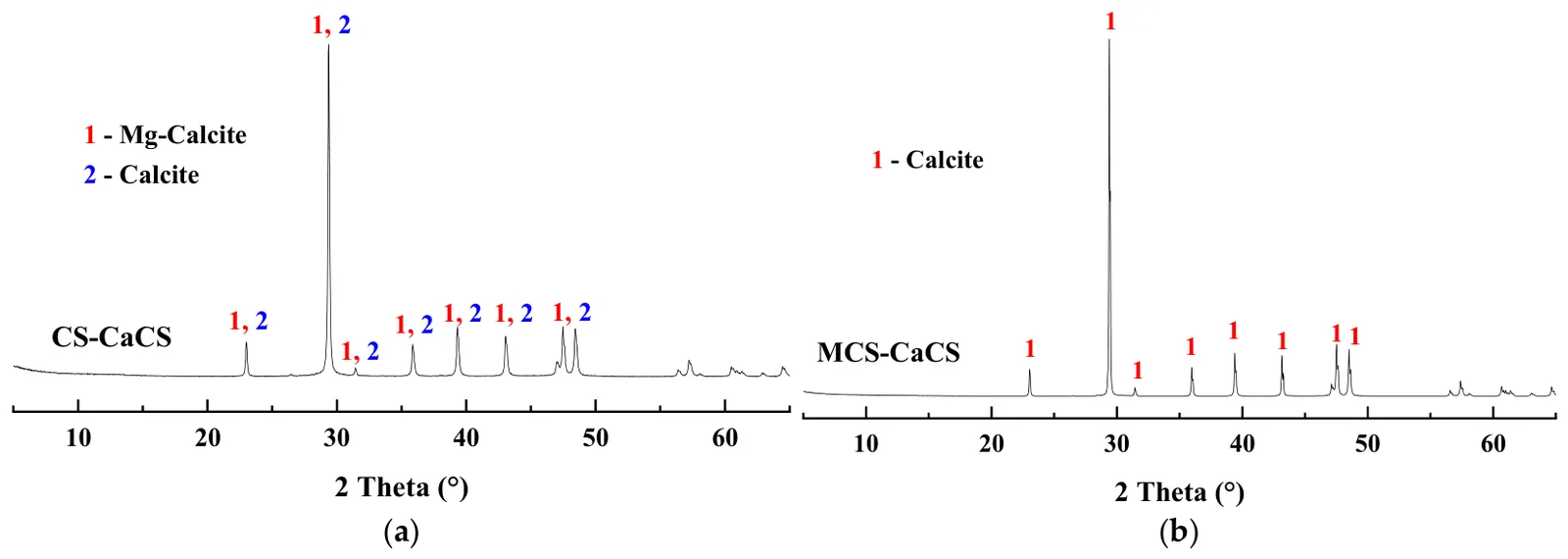
XRD patterns of (**a**) CS-derived CaCO_3_ and (**b**) MCS-derived CaCO_3_.

Carbonation of carbide slag proceeds in a multi-ionic environment containing residual alkalis, sulfates, and multivalent cations (notably Mg^2+^, Sr^2+^, Ba^2+^), which remain present after carbonation and/or are partially incorporated into the precipitated carbonate phase (Table 3). Such ions alter carbonate precipitation through changes in ion activity and complexation equilibria, thereby shifting nucleation thresholds and growth rates [34]. In particular, Mg-bearing species can enter the calcite lattice via substitution and promote Mg-substituted calcite formation, which manifests as impurity-related phase features in XRD (Figure 6) and is widely associated with lattice distortion and defect generation [35,36]. These impurity-driven deviations lead to a carbonate product with perturbed crystallographic regularity and modified surface termination chemistry, both of which are critical determinants of interfacial interactions in aqueous cementitious environments [37,38].

(2) Unregulated nucleation–growth kinetics and templating from the carbide slag

Beyond solution chemistry, the parent carbide slag provides a non-uniform physical template: particle size and morphology vary markedly, producing spatially uneven nucleation-site density and surface energy landscapes (Figure 2). Under such conditions, carbonation tends to proceed through localized supersaturation and burst-like precipitation, yielding CaCO_3_ with broad size dispersion, irregular aggregates, and non-equilibrium morphologies (consistent with the SEM morphology of CS-derived CaCO_3_ shown in Figure 6) [39,40]. This is not merely an “activity” issue. Kinetically uncontrolled precipitation promotes (i) agglomerate-dominated packing, (ii) reduced accessible surface area for productive hydrate attachment, and (iii) a mismatch in interfacial energy relative to the hydrating cement pore solution [41]. Collectively, these effects favor discontinuous hydrate deposition around carbonate agglomerates rather than close particle–hydrate association [42].

In combination, impurity-driven crystallographic deviation and kinetically uncontrolled precipitation produce CS-derived CaCO_3_ surfaces that are morphologically less capable of establishing a continuous, load-bearing CaCO_3_-hydrate interface. This provides a mechanistic basis for the interfacial gaps and discontinuities observed in the CS-CaCS system (Section 3.1) and anticipates the need for regulation strategies that simultaneously (i) suppress impurity inheritance and (ii) steer carbonation toward controlled nucleation–growth behavior.

#### 3.2.2. Regulation of Carbonation Toward MCS-Derived CaCO_3_

(1) Regulation of carbonation chemistry through impurity suppression

To mitigate impurity-induced perturbations during carbonate formation, acid washing was applied before wet milling and carbonation. This operation was intended to reduce the transfer of soluble alkalis, sulfate-containing species, and weakly bound multivalent ions from raw carbide slag into the carbonation environment. The resulting changes in the chemical composition of the carbonation products and the dissolved-ion concentrations are summarized in Table 3.

Compared with CS-CaCS, the MgO and SO_3_ contents of MCS-CaCS decreased from 0.93% and 1.52% to 0.33% and 0.51%, corresponding to reductions of approximately 64.5% and 66.4%, respectively. The dissolved Sr and S concentrations likewise decreased from 19.0 and 7.9 mg/L to 7.72 and 2.8 mg/L, respectively. These compositional changes demonstrate that the integrated pretreatment route substantially suppressed the transfer of impurity species into the subsequent carbonation process. Because MCS-CaCS was produced using the complete treatment route, these reductions represent the overall suppression of impurity inheritance and should not be interpreted as the independently quantified removal efficiency of acid washing alone.

As shown in Figure 6, calcite was the dominant crystalline phase in both CS-CaCS and MCS-CaCS, and no residual portlandite was detected in either product. The absence of detectable portlandite indicates near-complete carbonation within the detection limit of XRD. The (104) reflection is compared with the reference positions of calcite (PDF#47-1743) and Mg-rich calcite (PDF#43-0697). Because Mg-bearing calcite forms a continuous solid solution, its reflections remain close to those of pure calcite, particularly at the relatively low Mg contents present in the current samples. Mg incorporation is therefore expressed primarily as a small shift or asymmetry of the calcite reflections rather than as a clearly separated diffraction peak.

The CS-CaCS pattern exhibits more pronounced broadening and asymmetry of the (104) reflection, whereas the corresponding reflection in MCS-CaCS is sharper and more symmetric. Consistently, the bulk Mg/Ca molar ratio calculated from the XRF data decreased from 0.0162 in CS-CaCS to 0.0056 in MCS-CaCS. These results support reduced Mg-associated perturbation of the calcite structure after the integrated treatment. However, because the bulk Mg/Ca ratio includes all Mg- and Ca-bearing components, it is not interpreted as a direct quantitative measurement of Mg occupancy in the calcite lattice.

(2) Regulation of precursor dispersion and carbonate crystallization

Following impurity suppression, wet milling and TEA addition were used to regulate different stages of carbonate formation. Wet milling was conducted before carbonation to disintegrate coarse precursor agglomerates and produce a more physically uniform feed suspension. This operation primarily modified the dispersion state of the calcium-bearing precursor but did not remove the soluble impurities present in the carbonation environment. By contrast, TEA was introduced during carbonation as a crystallization regulator. Through its complexation with dissolved Ca species and adsorption on growing carbonate surfaces, TEA can modify the nucleation and growth behavior of CaCO_3_ [32]; however, it does not replace the preceding removal of soluble impurities or the mechanical disintegration of precursor agglomerates.

**Figure 7:**
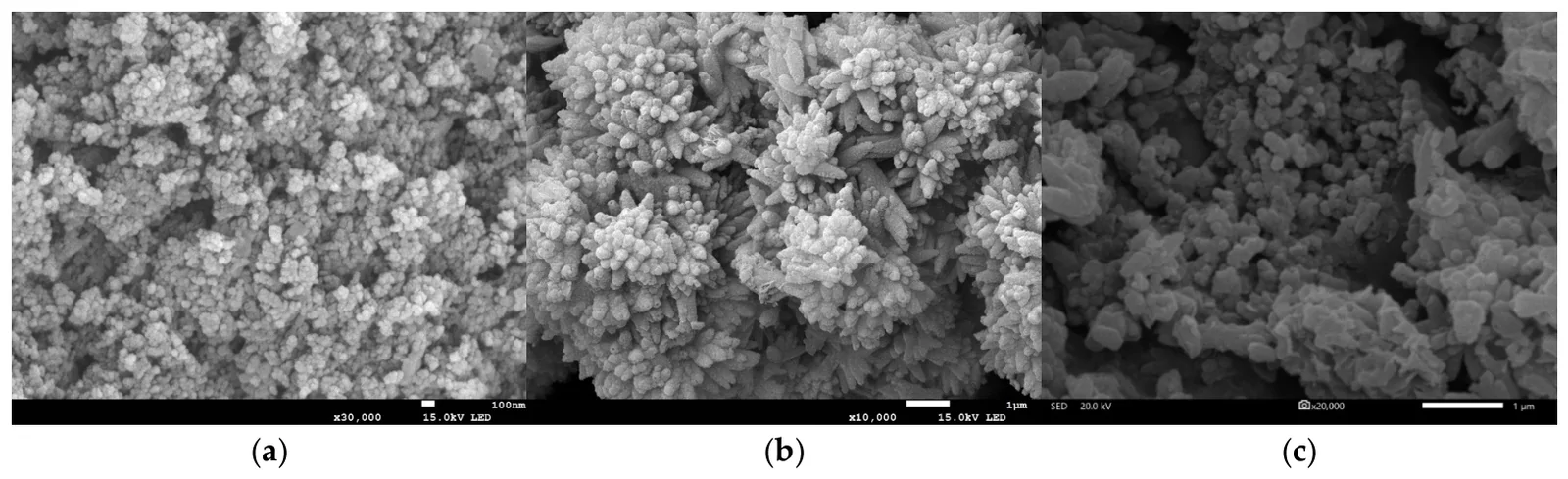
SEM morphology of CaCO_3_ in (**a**) CH-CaCS, (**b**) CS-CaCS and (**c**) MCS-CaCS.

The two operations therefore served different and complementary purposes: wet milling regulated the physical state of the precursor before carbonation, whereas TEA acted during carbonate precipitation. Following the integrated treatment, the resulting MCS-derived CaCO_3_ exhibited fewer coarse and irregular clusters and a more compact particle morphology than the untreated CS-derived product (Figure 7). These observations demonstrate the overall effect of the integrated route but do not independently quantify the contribution of wet milling or TEA.

(3) Changes in apparent dynamic wetting behavior

**Figure 8:**
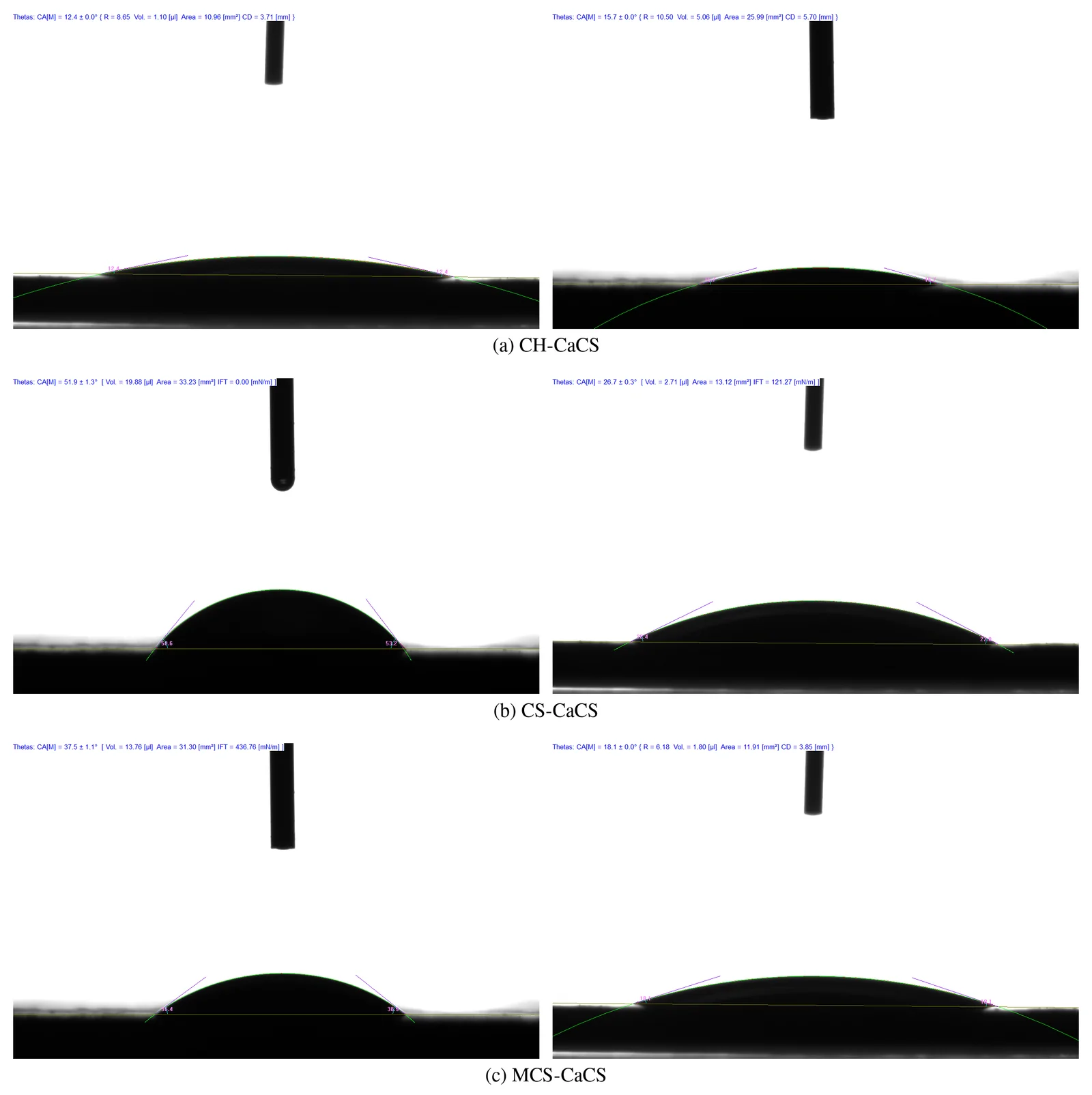
Contact-angle evolution of different carbonated calcium suspensions: (**a**) CH-CaCS, (**b**) CS-CaCS, and (**c**) MCS-CaCS.

Dynamic contact-angle measurements were used to compare the apparent wetting responses of CH-CaCS, CS-CaCS, and MCS-CaCS (Figure 8). CH-CaCS exhibited the lowest initial contact angle, whereas CS-CaCS showed the highest value, indicating greater resistance to spreading immediately after deposition. Following the integrated treatment, the initial contact angle of MCS-CaCS decreased markedly relative to CS-CaCS and approached that of CH-CaCS. This change indicates that the modified suspension established contact with the test surface more readily during the initial stage of spreading.

The contact angles of all three suspensions subsequently decreased between 0 and 5 s, reflecting continued dynamic spreading. The initial contact angle characterizes the resistance encountered immediately after droplet deposition, whereas its subsequent decrease describes the short-time spreading response rather than an equilibrium wetting state. The lower initial value and enhanced spreading response of MCS-CaCS indicate that the integrated treatment improved the apparent wettability of the carbide-slag-derived suspension. Together with the reduced impurity content and modified particle morphology described above, this result provides further evidence that the physicochemical characteristics of CS-CaCS were effectively modified, thereby favoring more extensive contact with cement particles during mixing and subsequent hydration.

### 3.3. From Interfacial Failure to Recovery in the MCS-CaCS System

Section 3.2 showed that pretreatment modifies the carbonation pathway of carbide slag, producing a CaCO_3_ phase with reduced impurity inheritance and improved structural regularity. This section examines how these modified carbonate characteristics manifest during cement hydration and subsequent microstructural and mechanical development.

#### 3.3.1. Characteristics of CaCO_3_ Participation in Cement Hydration

**Figure 9:**
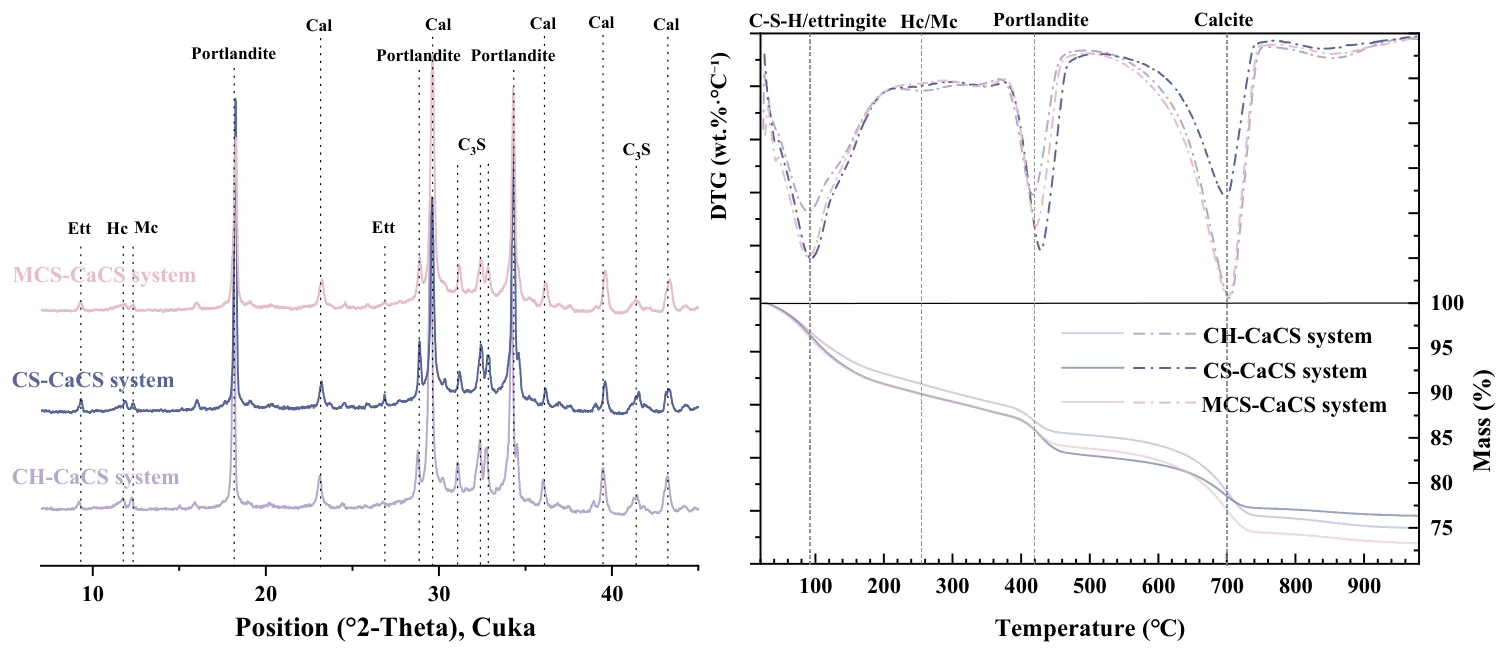
Experimentally determined phase assemblages of CH-CaCS, CS-CaCS, and MCS-CaCS systems (at 28 days, w/b = 0.35).

Figure 9 compares the XRD and TG–DTG results of the CH-CaCS, CS-CaCS, and MCS-CaCS pastes at 28 days. Although all three systems contain the principal hydration products of Portland cement, clear differences remain in the relative development of the sulfate- and carbonate-bearing aluminate phases [43,44].

The CH-CaCS paste exhibits distinct reflections associated with hemicarboaluminate (Hc) and monocarboaluminate (Mc), consistent with the reaction of readily available CaCO_3_ with the aluminate phases of cement. By contrast, CS-CaCS retains a more pronounced ettringite (AFt) reflection and shows weaker Hc/Mc-related features, indicating a more limited AFt-to-carbonate-AFm conversion. After the integrated treatment, MCS-CaCS exhibits enhanced Hc- and Mc-related reflections together with a reduced predominance of AFt. Its phase assemblage therefore shifts toward that of CH-CaCS, although the two systems do not exhibit identical hydration behavior.

**Figure 10:**
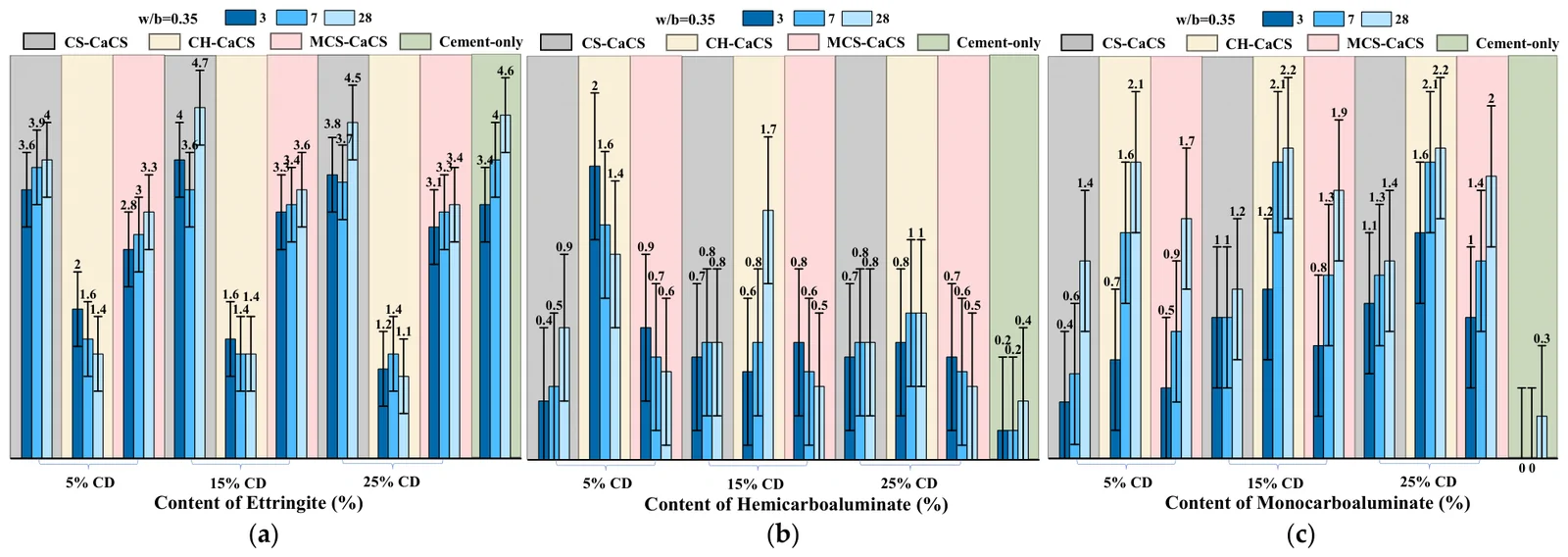
Hydration evolution of cement pastes incorporating CaCS at different carbonate dosages: (**a**) Ettringite (AFt), (**b**) Hemicarboaluminate (Hc), and (**c**) Monocarboaluminate (Mc).

The TG–DTG curves provide complementary information on the hydrated phase assemblages. The low-temperature mass-loss region is associated mainly with dehydration of C–S–H, AFt, and AFm-type phases, while the dehydroxylation of portlandite and decarbonation of CaCO_3_ occur in the intermediate- and high-temperature regions, respectively. The calcite-related decarbonation feature is less pronounced in CS-CaCS than in MCS-CaCS. However, the position and shape of the decarbonation feature depend on the carbonate phase and thermal-analysis conditions [45]; this feature was therefore not used independently to quantify carbonate participation in hydration. Instead, carbonate–aluminate participation was evaluated using the phase-evolution indices presented in Figure 10.

The XRD-derived phase-evolution indices confirm that the differences observed at 28 days result from distinct phase-evolution pathways rather than from an isolated diffraction feature. Across the investigated curing ages and carbonate dosages, CS-CaCS generally retains a stronger AFt-related response and develops lower Hc- and Mc-related indices than CH-CaCS. Across the investigated curing ages and carbonate dosages, CS-CaCS generally retains a stronger AFt-related response and develops lower Hc- and Mc-related indices than CH-CaCS. Compared with CS-CaCS, MCS-CaCS exhibits lower AFt-related indices together with increased Hc- and Mc-related indices, indicating greater participation of the modified carbonation product in the development of carbonate-bearing AFm phases [46,47].

This recovery is nevertheless partial. The Hc/Mc evolution of MCS-CaCS approaches, but does not completely reproduce, that of CH-CaCS. The remaining difference indicates that the behavior of the carbonation product is influenced not only by the nominal carbonate dosage but also by the chemical and physical characteristics inherited from the calcium source and preparation route.

**Figure 11:**
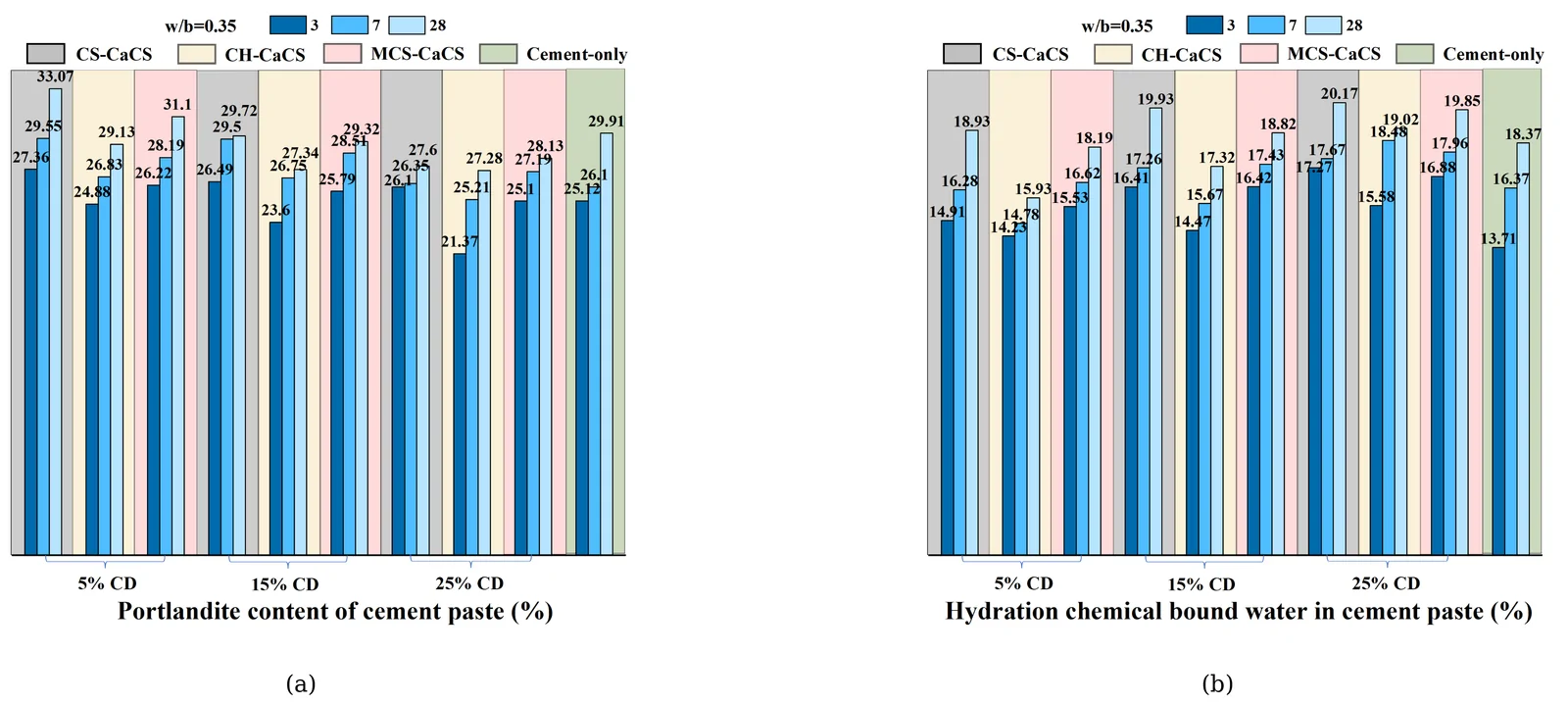
Thermogravimetric-derived hydration indices of cement pastes incorporating CaCS: (**a**) Portlandite Content and (**b**) Chemically Bound Water.

Figure 11 compares the TG-derived portlandite content and chemically bound water of the three cement-paste systems. These two indices provide an overall assessment of the extent of cement hydration but do not follow the same ranking as compressive strength. In particular, CS-CaCS can exhibit relatively high portlandite and chemically bound water (CBW) values while retaining lower mechanical performance than MCS-CaCS and CH-CaCS. The inferior strength of CS-CaCS therefore cannot be explained solely by a lower overall degree of cement hydration.

Taken together, the XRD and TG results show that the integrated treatment alters the manner in which CS-derived CaCO_3_ participates in cement hydration, particularly by promoting carbonate-bearing AFm formation. However, the bulk hydration indices alone do not fully explain the differences in mechanical performance. The accompanying changes in particle–hydrate organization and local mechanical response are therefore examined in Section 3.3.2 and Section 3.3.3.

#### 3.3.2. Microstructural Features Associated with CaCO_3_–Cement Interaction

**Figure 12:**
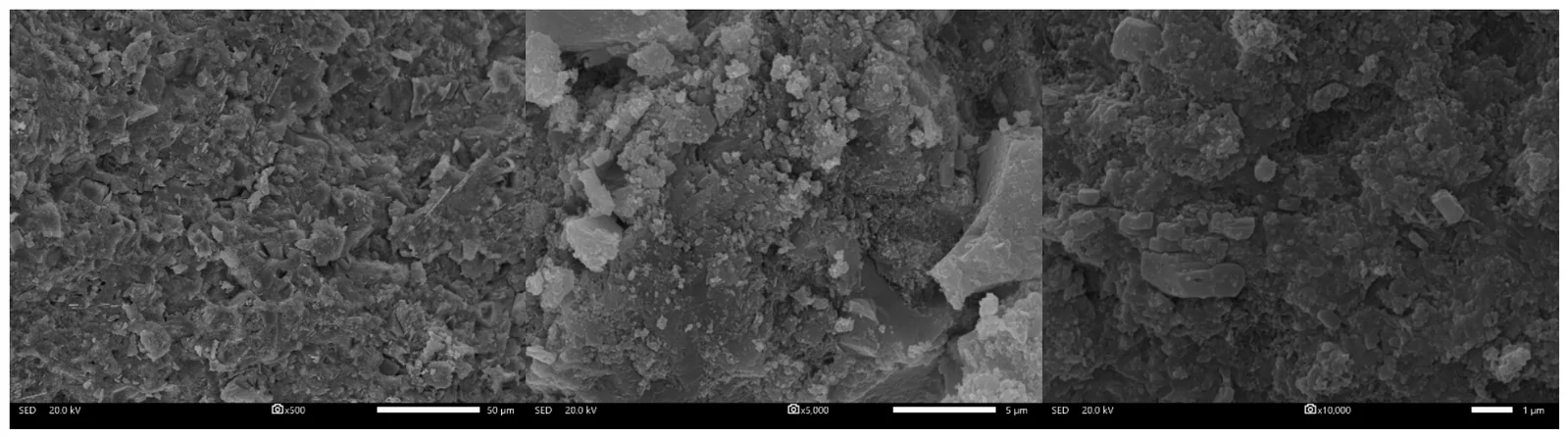
Micromorphology of Cement Paste Incorporating MCS-CaCS.

The microstructural consequences of the integrated treatment were further examined using fracture-surface SEM at multiple magnifications. Figure 12 presents the MCS-CaCS paste at three magnifications, allowing its overall spatial morphology and local particle–hydrate contact features to be examined. At 5000× magnification, the paste shows fewer coarse, loosely packed clusters and fewer large open regions associated with particle agglomeration than those observed in CS-CaCS. At 10,000× magnification, the modified carbonation products appear more closely surrounded by hydration products, although local morphological heterogeneity remains. Compared with the untreated CS-CaCS system shown in Figure 4b, these observations indicate that the integrated treatment reduces the prevalence of coarse agglomerate-associated discontinuities and promotes closer spatial association between the carbonation products and the hydrated matrix.

#### 3.3.3. Local Mechanical Response Associated with the Integrated Treatment

**Figure 13:**
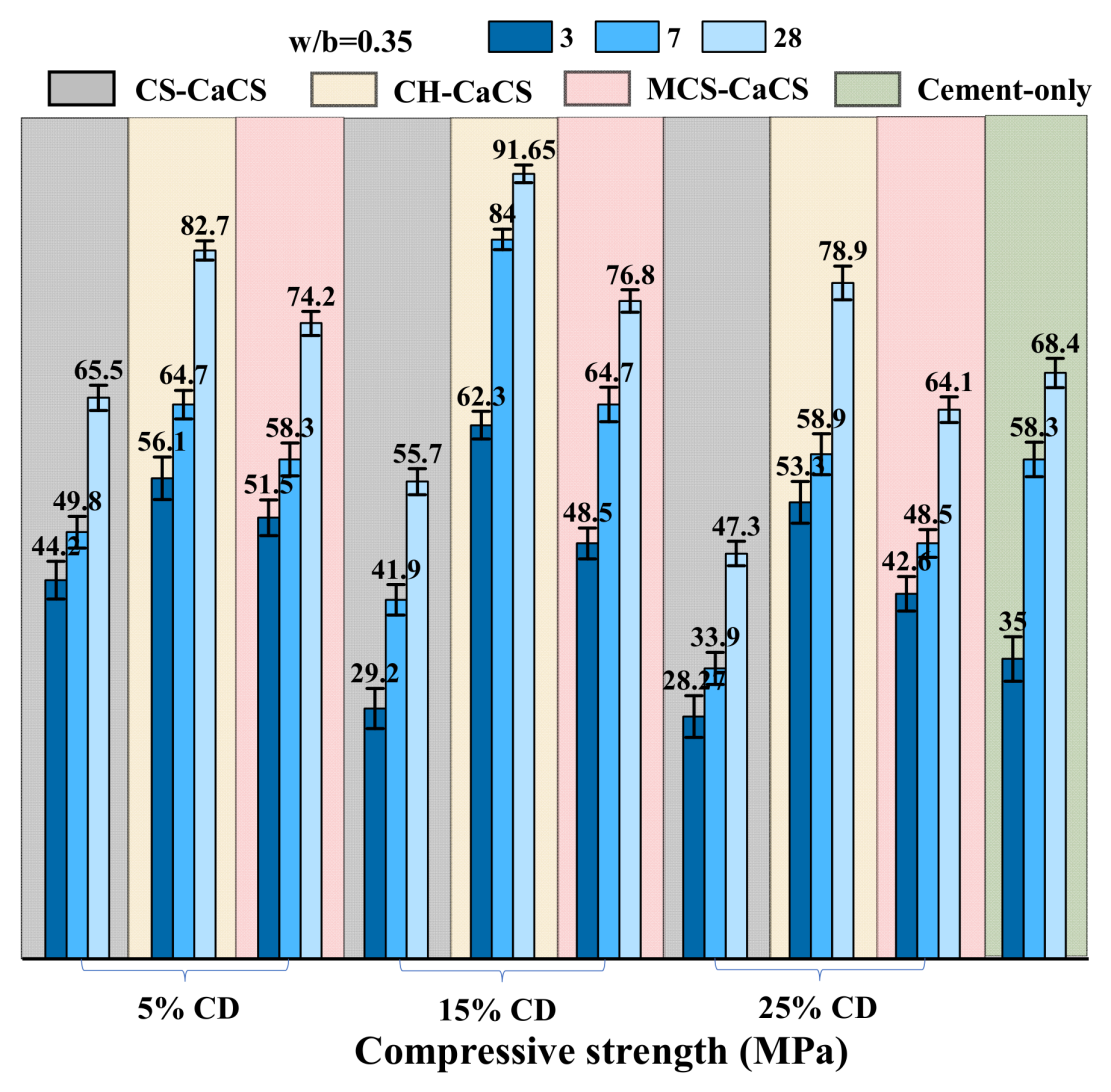
Compressive strength of CH-CaCS, CS-CaCS, and MCS-CaCS systems.

As shown in Figure 13, incorporation of MCS-CaCS leads to a substantial recovery of compressive strength associated with CS-derived CaCO_3_, while remaining below the CH-CaCS reference. For instance, at 15% CD and 28d, the strength of CS-CaCS decreases to approximately 55.7 MPa, whereas MCS-CaCS recovers to about 76.8 MPa, compared with approximately 91.7 MPa for CH-CaCS. This trend indicates that pretreatment mitigates the mechanical inefficiency associated with CS-derived CaCO_3_.

**Figure 14:**
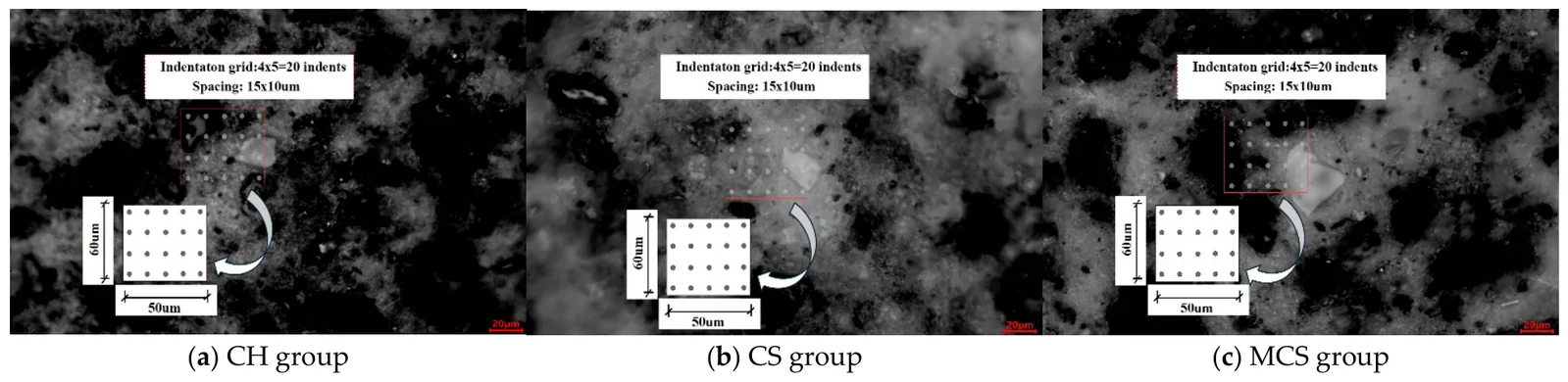
Schematic illustration of the nanoindentation grid across the CaCO_3_–cement interface. (The curved arrows link the selected indentation regions to schematic representations of the indentation grids; the dots indicate individual indentation positions).

To examine the local mechanical changes accompanying the recovery in compressive strength, nanoindentation was performed across carbonate-adjacent regions of the three cement-paste systems [48]. Figure 14 illustrates the location and arrangement of the nanoindentation grids within these regions. The measured response represents the combined contribution of the carbonation products, surrounding hydration products, local pores, and their contact regions.

As summarized in Table 4, CS-CaCS exhibits the lowest mean hardness and reduced modulus among the three systems, indicating that compliant regions account for a substantial proportion of the examined area. MCS-CaCS shows higher mean hardness and reduced modulus than CS-CaCS, consistent with the emergence of mechanically stiffer local domains following the integrated treatment. Nevertheless, the standard deviations and coefficients of variation remain high and, for several parameters, exceed those of CS-CaCS. The treatment therefore did not produce a mechanically homogeneous region. Instead, it altered the range and relative occurrence of local mechanical responses while substantial spatial heterogeneity remained.

**Figure 15:**
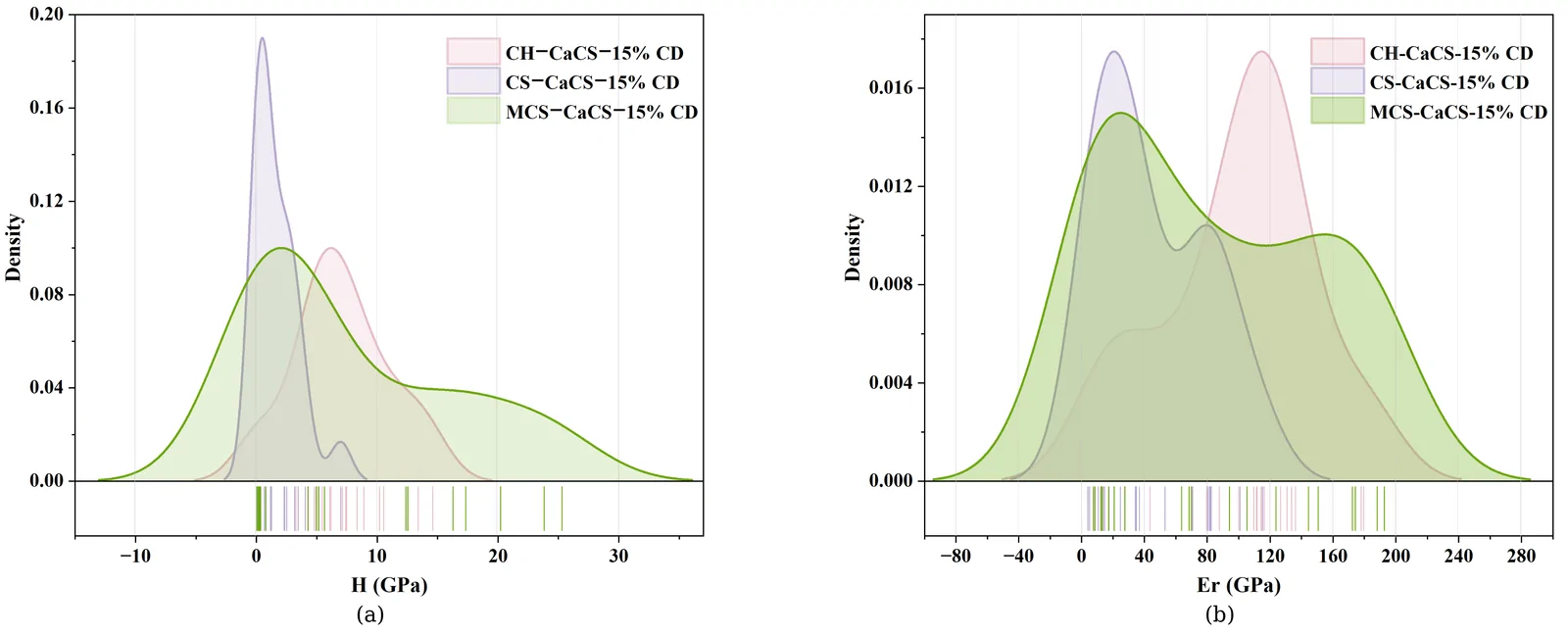
Kernel Density Estimates (KDE) of (**a**) Hardness and (**b**) Reduced Modulus of CH-CaCS, CS-CaCS, and MCS-CaCS systems.

This interpretation is supported by the KDE profiles in Figure 15. The CS-CaCS distributions are concentrated predominantly in the low-hardness and low-modulus ranges, although higher-value responses also occur locally. By contrast, the MCS-CaCS distributions extend more clearly toward the range measured for CH-CaCS while retaining a low-value population. The broad and apparently bimodal distributions indicate the coexistence of mechanically compliant and stiff local domains rather than a uniform shift in the entire examined region. Accordingly, the increase in the mean response of MCS-CaCS reflects the development of a greater high-value contribution, not the elimination of local heterogeneity.

**Figure 16:**
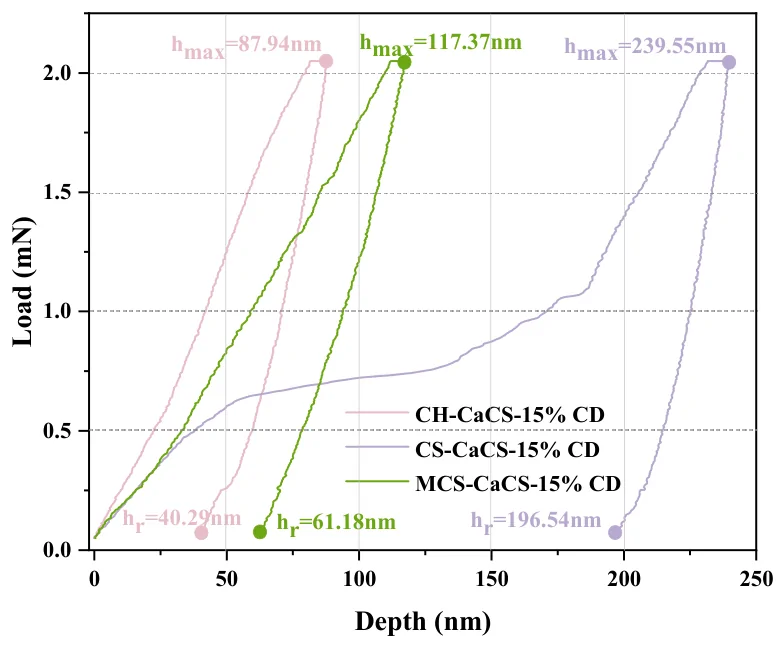
Representative Load–Depth (P–h) Curves of CH-CaCS, CS-CaCS, and MCS-CaCS systems.

The representative load–depth curves in Figure 16 further illustrate the variability within each system. The complete load–depth datasets for the three systems are provided as Supplementary Data. CS-CaCS contains a greater proportion of curves characterized by large maximum penetration depths and substantial residual depths after unloading, whereas MCS-CaCS includes more curves with lower penetration depths and greater unloading recovery. The irreversible component of deformation is quantified by the residual-depth ratio (h_r_/h_max_) and plastic-work fraction (W_p_/W_tot_) in Table 4. Higher values of these parameters indicate a larger retained deformation and a greater proportion of indentation work dissipated plastically. Their reduction from CS-CaCS toward the CH-CaCS range in MCS-CaCS therefore supports an increased contribution from locally stiffer and more recoverable domains, although the broad distributions confirm that compliant regions remain present.

The origin of these local responses cannot be assigned to a single mechanism from the load–depth curves alone. Large penetration and residual depths may arise from deformation of a porous or weakly hydrated matrix, local pore collapse, particle–matrix sliding or debonding, and, where the indenter directly encounters a carbonate agglomerate, particle damage or crushing. Conversely, the higher hardness and modulus recorded in parts of MCS-CaCS may reflect changes in the morphology and mechanical response of the carbonation products, closer association with hydration products, and/or a higher local degree of hydration in the adjacent matrix.

The TG-derived portlandite and chemically bound water results do not follow the same ranking as strength, indicating that differences in the bulk degree of hydration alone are insufficient to explain the mechanical response. However, these bulk indices cannot exclude spatial differences in hydration around individual carbonation products. Particle-size refinement, reduced agglomeration, physical filling, and heterogeneous nucleation may also contribute to the local and macroscopic response. The nanoindentation results should therefore be interpreted as evidence that the integrated treatment redistributes the local mechanical population toward a greater contribution from stiff and recoverable domains, rather than as proof that strength recovery arises exclusively from intrinsic CaCO_3_ properties or from a uniformly restored interface.

## 4. Discussion

The results demonstrate that the performance of carbide-slag-derived CaCO_3_ in cement paste is governed by the combined effects of its composition, particle organization, hydration participation, and local mechanical response. Although CaCO_3_ is generally considered to influence cement hydration through physical filling, heterogeneous nucleation, and carbonate–aluminate reactions, these mechanisms alone do not fully explain the performance differences among CH-CaCS, CS-CaCS, and MCS-CaCS. In particular, the TG-derived portlandite content and chemically bound water do not follow the same ranking as compressive strength. Therefore, the mechanical response cannot be interpreted solely in terms of the bulk degree of cement hydration.

The untreated CS-CaCS system combines greater impurity inheritance, coarse and irregular particle agglomeration, a higher initial resistance to droplet spreading, and lower Hc- and Mc-related phase-evolution indices. Fracture-surface SEM further shows comparatively open regions around coarse particle clusters, while nanoindentation indicates that low-hardness and low-modulus responses account for a substantial part of the investigated regions. Taken together, these observations indicate that the untreated carbonation product is less effectively incorporated into the spatial organization of the hydrated matrix.

Following the integrated pretreatment–carbonation route, MCS-CaCS exhibits reduced MgO and SO_3_ contents, fewer coarse particle clusters, a lower initial contact angle, and increased Hc- and Mc-related indices relative to CS-CaCS. These changes are accompanied by increases in mean nanoindentation hardness and reduced modulus and by substantial recovery of compressive strength. Nevertheless, the relatively high coefficients of variation and the broad distributions containing both low- and high-value responses show that MCS-CaCS remains mechanically heterogeneous. The treatment therefore increases the contribution of mechanically stiff local regions.

Several concurrent mechanisms may contribute to this performance recovery. First, reduced coarse agglomeration may decrease agglomerate-associated defects and improve the physical packing of the carbonation products. Second, the modified particle organization and short-time spreading response may increase the accessibility of the dispersed carbonation products to the aqueous cement environment, potentially providing more spatially distributed sites for heterogeneous nucleation and hydrate growth. Third, the increased Hc- and Mc-related indices indicate greater participation of MCS-derived CaCO_3_ in carbonate–aluminate reactions. The present results are consistent with the combined contribution of physical filling, heterogeneous nucleation, carbonate–aluminate reaction, and modified local particle–hydrate organization. However, particle-size distribution, packing efficiency, nucleation kinetics, and CaCO_3_ dissolution rates were not measured independently; the relative contribution of each mechanism therefore cannot be quantified from the present dataset.

The nanoindentation results should likewise be interpreted as a local composite response rather than as the intrinsic mechanical properties of the CaCO_3_ particles. Large maximum and residual penetration depths may result from a porous or weakly hydrated matrix, local pore collapse, particle-matrix sliding or debonding, or deformation of particle agglomerates. Conversely, the higher hardness and reduced modulus measured in parts of MCS-CaCS may reflect closer particle–hydrate association, differences in local hydration, modified particle morphology, or a combination of these effects. Consequently, the nanoindentation results support a redistribution toward a greater contribution from stiff and recoverable local regions.

Compared with previous approaches that use carbide slag directly as an alkaline activator, clinker raw material, or cementitious component, the present route first converts the calcium-bearing residue into a dispersed carbonate suspension. The results show that the cementitious performance of waste-derived CaCO_3_ cannot be evaluated from carbonate content alone. The precursor composition and carbonation pathway influence particle agglomeration, apparent spreading behavior, hydration participation, and the resulting local mechanical response. The integrated pretreatment–carbonation route therefore provides a potential pathway for producing a more cement-compatible carbonate suspension from carbide slag, although its process efficiency, environmental benefit, and scalability require further evaluation.

Several limitations should be considered when interpreting these findings. Acid washing, wet milling, and TEA-assisted carbonation were implemented sequentially as an integrated route, and their individual and interactive contributions were not isolated experimentally. The fracture-surface SEM images and short-time contact-angle measurements provide qualitative information on local morphology and apparent dynamic spreading, respectively, but do not quantify interfacial-gap statistics, bulk pore structure, or equilibrium wetting behavior. Future work should therefore combine stepwise treatment groups, quantitative particle-size and pore-structure characterization, polished-section or three-dimensional imaging, and direct measurements of nucleation and CaCO_3_ dissolution kinetics.

## 5. Conclusions

The mechanical inefficiency of cement systems incorporating CaCO_3_ precipitated from untreated carbide slag was associated with impurity inheritance, irregular particle aggregation, less favorable initial wetting behavior, limited carbonate–aluminate participation, and a predominance of mechanically compliant local domains. These results demonstrate that the formation pathway of carbide-slag-derived CaCO_3_ strongly influences its subsequent interaction with the hydrating cement matrix.

The integrated pretreatment–carbonation route suppressed impurity inheritance and modified the particle and crystallographic characteristics of the carbonation product. The MgO and SO_3_ contents decreased from 0.93% and 1.52% in CS-CaCS to 0.33% and 0.51% in MCS-CaCS, respectively. At 15% CD and 28 days, the compressive strength increased from 55.7 MPa for CS-CaCS to 76.8 MPa for MCS-CaCS, representing an improvement of approximately 37.9%, although it remained below the 91.7 MPa obtained for CH-CaCS. The mean nanoindentation hardness increased from 1.81 to 6.18 GPa, while the mean reduced modulus increased from 50.0 to 74.7 GPa. Nevertheless, the relatively high coefficients of variation and broad KDE distributions show that MCS-CaCS remained mechanically heterogeneous, with compliant and stiff local domains coexisting within the investigated regions.

Overall, the performance recovery of MCS-CaCS is attributed to the concurrent effects of reduced impurity inheritance and particle agglomeration, improved surface accessibility, enhanced carbonate participation in hydration, and an increased contribution from mechanically stiff local domains. The integrated pretreatment–carbonation route therefore provides a viable approach for converting carbide slag into a more cement-compatible carbonate suspension and informs the development of calcium-rich industrial residues as functional additives for low-carbon cementitious materials.

## Figures and Tables

**Table 1:** Chemical and mineral compositions of Portland cement and carbide slag (%).

	Al_2_O_3_ ^a^	CaO ^a^	SiO_2_ ^a^	Fe_2_O_3_ ^a^	MgO ^a^	K_2_O ^a^	Na_2_O ^a^	SO_3_ ^a^	C_3_S ^b^	C_3_A ^b^	C_2_S ^b^	C_4_AF ^b^	Gp ^b^	Portlandite ^b^	Calcite ^b^
PC	3.61	61.46	19.38	3.285	3.17	0.858	0.189	3.21	67.8	7.8	6.4	7.5	3.2	-	-
Raw-CS	1.41	78.27	3.05	1.285	3.17	0.858	0.189	2.217	-	-	-	-	-	97.3	2.6

Note: ^a^ Chemical composition determined by XRF and expressed as oxide equivalents; ^b^ phase contents determined by XRD–Rietveld refinement.

**Table 2:** Mix Proportions for Slurry Pastes.

Sample ID	w/b	CD (%)	Cem (g)	CH (g)	CS/MCS (g)	H_2_O_susp_ (g)	Mixing Cal_susp_ (g)	Mixing Water (g)
PC	0.35	-	700	-	-	-	-	245
CS-CaCS-5	0.35	5	700	-	60	600	300	-
CS-CaCS-15	0.35	15	760	-	140	600	450	-
CS-CaCS-25	0.35	25	750	-	200	600	600	-
CH-CaCS-5	0.35	5	700	60	-	600	300	-
CH-CaCS-15	0.35	15	760	140	-	600	450	-
CH-CaCS-25	0.35	25	750	200	-	600	600	-
MCS-CaCS-5	0.35	5	700	-	60	600	300	-
MCS-CaCS-15	0.35	15	760	-	140	600	450	-
MCS-CaCS-25	0.35	25	750	-	200	600	600	-

Note: CD: the CaCO_3_ dosage (% by mass); Cem: cement; CH: Ca(OH)_2_ in suspension; CS/MCS: mass of untreated or pretreated carbide slag used to prepare the suspension, respectively; H_2_Osusp: Water in initial suspension; Mixing Cal_susp_: Calcite suspension usage; Mixing water: deionized water added separately during paste preparation.

**Table 3:** Elemental composition (%) and ionic concentrations (mg/L) of MCS-CaCS (XRF and ICP analysis).

	Raw-CS	CS-CaCS	MCS-CaCS		Raw-CS	CS-CaCS	MCS-CaCS
Al_2_O_3_ ^a^	1.41	1.02	0.9	Ca ^b^	987.6	297.7	306.4
CaO ^a^	78.27	80.11	82.5	Mg ^b^	0.14	0.007	0.004
SiO_2_ ^a^	3.05	3.7	3.2	Ba ^b^	5.8	3.1	1.7
Fe_2_O_3_ ^a^	1.285	1.22	1.14	K ^b^	1.9	2.1	1.3
MgO ^a^	3.17	0.93	0.33	Na ^b^	4.8	5.1	1.86
K_2_O ^a^	0.858	0.7	0.36	S ^b^	94.1	7.9	2.8
Na_2_O ^a^	0.189	0.11	0.04	Si ^b^	1.3	0.24	0.38
SO_3_ ^a^	2.217	1.52	0.51	Sr ^b^	87	19	7.72

Note: ^a^ obtained by XRF, ^b^ obtained by ICP–OES.

**Table 4:** Summary of Nanoindentation-Derived Mechanical Parameters of CH-CaCS, CS-CaCS, and MCS-CaCS systems.

Group	CH-CaCS	CS-CaCS	MCS-CaCS
H_mean_ (GPa)	7.81	1.809	6.18
H_std_ (GPa)	3.278	1.81	7.06
H_CV_	0.42	1	1.142
Er_mean_ (GPa)	109.458	50.01	74.671
Er_std_ (GPa)	39.006	33.706	58.592
Er_CV_	0.356	0.674	0.785
(hr/hmax)_mean_	0.728	0.893	0.789
(hr/hmax)_std_	0.075	0.058	0.136
(hr/hmax)_CV_	0.103	0.065	0.172
(H/Er)_mean_	0.076	0.03	0.059
(H/Er)_std_	0.029	0.016	0.036
(H/Er)_CV_	0.381	0.517	0.621
(Wp/Wtot)_mean_	0.527	0.741	0.587
(Wp/Wtot)_std_	0.123	0.097	0.223
(Wp/Wtot)_CV_	0.233	0.131	0.38

## Data Availability

The original contributions presented in this study are included in the article/Supplementary Materials. Further inquiries can be directed to the corresponding author.

## Supplementary Materials

The following supporting information can be downloaded at: https://www.mdpi.com/article/10.3390/ma19173765/s1. Nanoindentation_Data: Raw nanoindentation data for the CH-CaCS, CS-CaCS, and MCS-CaCS cement pastes.
